# Treatment options for long head of biceps tendon tenodesis

**DOI:** 10.3389/fsurg.2026.1753862

**Published:** 2026-04-08

**Authors:** Bo Wang, Meng Wu, Yongqiang Zhang, Yalong Yang, Yang Li, Junliang Liu, Shouhu Mi, Yong Shen, Zhao Zhang

**Affiliations:** Department of Joint Surgery and Sports Medicine, Norinco General Hospital, Xi'an, China

**Keywords:** arthroscopy, long head of biceps tendon (LHBT), non-subpectoral, subpectoral, surgical technique, tenodesis

## Abstract

The long head of the biceps tendon (LHBT) is frequently described as a primary “pain generator” in anterior shoulder pain. While both tenotomy and tenodesis are commonly employed for LHBT pathologies, no consensus exists on the optimal procedure, as neither has demonstrated clear superiority. It is widely recognized, however, that tenotomy is associated with a risk of “Popeye” deformity and aesthetic concerns, whereas tenodesis mitigates these issues. The tenodesis literature reveals a variety of LHBT fixation methods, which differ primarily in the location of fixation, the choice of implants, and the suturing technique. This article classifies these techniques into two main groups (subpectoral and non-subpectoral tenodesis) to provide an overview of the surgical techniques and to contrast their advantages and disadvantages, thereby assisting surgeons in individualized decision-making based on factors such as tendon quality, bone density, economic considerations, injury type, cosmetic concerns, and surgeon experience.

## 1 Introduction

The long head of the biceps brachii originates from the supraglenoid tubercle of the scapula, descends through the intertubercular sulcus, and finally inserts into the radial tuberosity. Its main functions are elbow flexion and forearm supination. The long head of the biceps tendon (LHBT) is often described as a primary “pain generator” for anterior shoulder pain (1, 2). Although LHBT pathology can occur in isolation, it is frequently associated with other shoulder conditions such as impingement syndrome, rotator cuff tears, and superior labrum anterior to posterior (SLAP) lesions. Common etiologies of LHBT pathology include SLAP lesions, tendinopathy, partial tears, and shoulder joint instability (3, 4). Tenotomy and tenodesis are the standard surgical approaches for LHBT pathology. However, there is a well-established consensus that tenotomy is significantly associated with the occurrence of the “Popeye” deformity and subsequent cosmetic concerns (5–7). A meta-analysis of five studies (227 cases each for tenotomy and tenodesis) found that tenodesis was superior to tenotomy in reducing the risk of the “Popeye” deformity, with 56 vs. 17 cases reported postoperatively (8).

Since Gilcreest first described the LHBT tenodesis in 1925 (9), the technique has undergone substantial evolution, with numerous modifications documented in the literature. Clinically, this procedure is indicated for a variety of pathological conditions. A systematic review reported that among patients undergoing LHBT tenodesis, the most common indications—which often overlapped—included partial tears (51%), tendon instability (49%), tenosynovitis (44%), SLAP tears (28%), and tendinopathy (26%) (1).

According to the literature (10–13), surgical approaches for LHBT tenodesis primarily include all-arthroscopic, arthroscopically assisted, and open procedures. Typically, open tenodesis requires initial arthroscopic examination and arthroscopic release of the proximal LHBT. The subsequent fixation site can be selected from the area extending from the articular margin at the apex of the intertubercular groove to the superior and inferior borders of the pectoralis major tendon. Gombera et al. reported that arthroscopic suprapectoral tenodesis and open subpectoral tenodesis yield comparable clinical outcomes and complication rates (14). However, a recent study has found that the complication rate of suprapectoral tenodesis is higher compared to subpectoral tenodesis (15). Although several biomechanical studies have analyzed different tenodesis techniques, robust clinical data demonstrating the superiority of one technique over others remain lacking (16, 17).

Currently, there remains a scarcity of literature that systematically summarizes and compares the technical details of LHBT tenodesis. Based on relevant studies, this review proposes a novel classification strategy by categorizing tenodesis techniques into subpectoral and non-subpectoral tenodesis, distinguished primarily by the location of fixation. Unlike previous reviews, this classification clarifies the general need for an auxiliary incision in subpectoral tenodesis, while the non-subpectoral category comprehensively covers all fixation sites spanning from the apex of the bicipital groove to the suprapectoral region, rather than being restricted to suprapectoral fixation alone. By systematically summarizing and comparing these techniques in terms of surgical methods, advantages, and limitations, this review aims to provide a comprehensive and clinically practical reference for surgeons in selecting individualized tenodesis approaches.

## 2 Subpectoral tenodesis

The subpectoral tenodesis procedure involves an initial arthroscopic exploration to confirm the diagnosis and manage any concomitant injuries. After transecting the LHBT proximally, the tenodesis is performed at the inferior border of the pectoralis major. This approach offers the advantage of demanding less technical expertise and enables the removal of most of the LHBT and its associated tendon sheath, which could theoretically lead to complete relief of symptoms caused by LHBT-related pathologies. Drawbacks include the need for an additional incision and the risks of proximal humerus fracture and brachial plexus injury (18–20). Notably, because the subpectoral location effectively releases the tendon sheath and the biceps tendon, thereby alleviating the patient's primary pain, it results in a lower reoperation rate compared to the suprapectoral tenodesis (21, 22).

### 2.1 Three-tunnel fixation technique

#### 2.1.1 Surgical procedure

**Figure 1:**
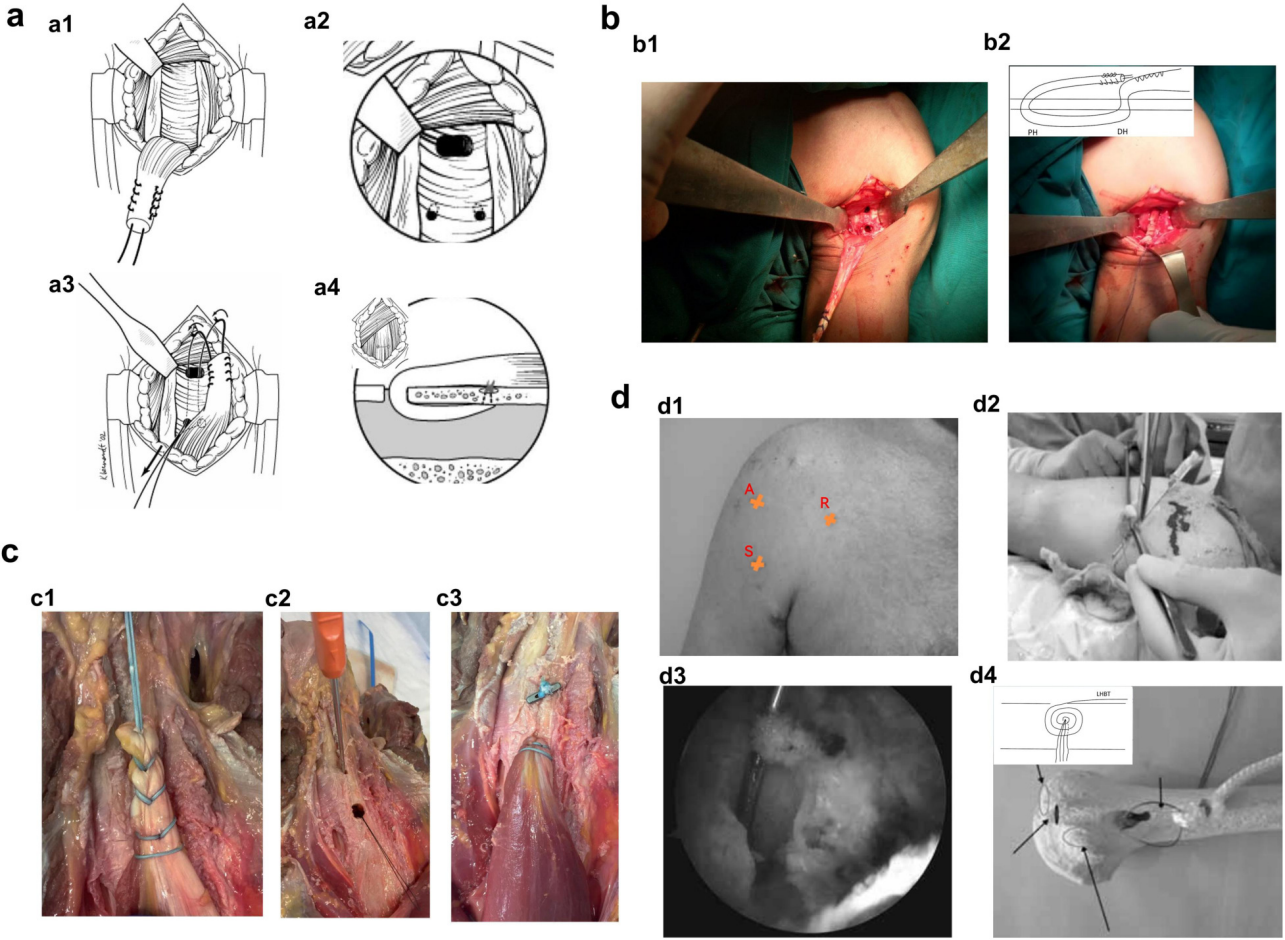
Diagram of the key steps for the three-tunnel fixation technique (**a**), double-tunnel fixation technique (**b**), unicortical suture button fixation technique (**c**), and keyhole proximal biceps tenodesis (**d**). (**a1**) Whipstitch suture of the proximal 15 mm of tendon. (**a2**) Creation of two spaced suture shuttle holes at the distal bicipital groove. (**a3**) Retrograde suture passage from distal holes to proximal trough, followed by retrieval and tying to the whipstitch. (**a4**) Final tendon fixation within the bone trough. Panel (**a**) is adapted from (23). (**b1**) Creation of proximal and distal bone tunnels in the anterior humeral cortex. (**b2**) Tendon fixation after shuttling through bone tunnels with tension adjustment. Panel (**b**) is adapted from (25). (**c1**) Tendon transected ∼2.5 cm proximal to myotendinous junction and sutured with No.2 FiberWire in modified whip-stitch, leaving tails at stump. (**c2**) Sequential drilling of a distal hollow tunnel (6–8 mm) and a proximal unicortical hole (2.5 mm), followed by suture shuttle retrieval. (**c3**) The tendon is docked and secured within the intramedullary tunnel using a suture button. Panel (**c**) is adapted from (26). (**d1**) Establishment of arthroscopic portals R, S, and A for keyhole proximal biceps tenodesis. (**d2**) A tendon plug is prepared by whip-stitching the tendon ∼2–2.5 cm distal to the myotendinous junction. (**d3**) The “keyhole” structure is completed by removing the bone bridge. (**d4**) The tendon plug is introduced and advanced using a suture shuttle technique. An audible “click” confirms secure locking into the keyhole. Panel (**d**) is adapted from (28).

This technique is based on the method described by Mazzocca et al. (24), utilizing a bone tunnel suture passage and an intracortical biceps tenodesis. The procedure is performed as follows (23):

(1) Transect the tendon 20–25 mm proximal to musculotendinous junction and trim excess tendon.
(2) Perform a whipstitch suture along the proximal 15 mm of the remaining tendon using a No.2 FiberWire (Figure 1a1).
(3) At the proximal bicipital groove just inferior to the pectoralis major tendon, create three 4.5 mm holes penetrating only the anterior cortex of the humerus. Connect these holes with a rongeur to form a bone trough.
(4) At the distal aspect of the bicipital groove, create two 3.2 mm sufficiently spaced suture shuttle holes to facilitate retrograde passage of a suture-passing device (Figure 1a2).
(5) Pass a suture passer retrograde from each distal holes, retrieve it from the proximal bone trough, and remove the device. Tie the ends of the sutures to the No.2 FiberWire and pull them out through the corresponding distal holes (Figure 1a3).
(6) Tension the FiberWire to advance the biceps tendon into the bone trough, then tie the two trailing sutures beneath the tendon to secure it in place (Figure 1a4).

#### 2.1.2 Advantages and limitations

This technique involves passing the tendon through a bone tunnel and securing it with tied suture tails, eliminating the need for additional implants. In our opinion, this reduces the economic burden on patients and avoids implant-related complications such as rejection and discomfort. We believe that by controlling the depth of tendon insertion into the bone tunnel, muscle tension can be precisely adjusted, which helps prevent postoperative muscle pain, promotes restoration of biceps morphology and function, and preserves muscle strength.

However, the technique also has certain limitations. Creating three bone tunnels in the bicipital groove may increase the risk of proximal humeral fracture. Furthermore, accidental penetration of the contralateral cortex during drilling may lead to nerve injury (24). Although this fixation method is relatively straightforward, Mazzocca et al. found that cyclic displacement of the bone tunnel was greater compared to fixation using interference screws or suture anchors (24).

### 2.2 Double-tunnel fixation technique

#### 2.2.1 Surgical procedure

Resect the thickened proximal portion of the LHBT, preserving 20–25 mm of the proximal myotendinous junction. The residual stump is then whip-stitched using No.2 FiberWire. Synovium is opened with an electrocautery knife to expose the bone of the biceps tendon tunnel (25).

Drill a 4.5 mm proximal bone tunnel through the anterior humeral cortex at the superior aspect of the exposed bone, then drill a second distal tunnel 15–20 mm inferiorly, at the level of the lower border of the pectoralis major (Figure 1b1) (25).

Subsequently, pass the FiberWire sutures retrograde from the distal tunnel using an arthroscopic suture hook—suture end first—and then pull them out through the proximal tunnel. The tendon is shuttled through both bone tunnels, and tension is adjusted by advancing the myotendinous junction to the distal tunnel. Finally, the tendon is woven and sutured onto itself (Figure 1b2) (25).

#### 2.2.2 Advantages and limitations

The authors believe that, similar to the three-tunnel fixation technique, the double-tunnel fixation technique also does not require the use of implants. Another advantage of this technique lies in its ability to precisely adjust the tension of the biceps tendon before final suture fixation and to potentially promote soft tissue healing. In addition, the technique is less technically demanding and is not associated with elevated risks of infection, nerve injury, or wound complications (25).

### 2.3 Unicortical suture button fixation technique

#### 2.3.1 Surgical procedure

After identifying biceps tendon pathology, the tendon is fixed with an epidural needle and arthroscopically released from its insertion. The arm is then placed in abduction and external rotation, and a 3–4 cm longitudinal incision is made along the course of the biceps tendon at the anteromedial aspect of the upper arm, just inferior to the lower border of the pectoralis major (26).

The aponeurosis overlying the pectoralis major and biceps tendons is incised to expose the biceps tendon. The myotendinous junction and its corresponding point on the humerus are identified and marked to serve as a reference for tension adjustment during fixation. The fixation needle is then removed, and the tendon is delivered out through the incision (26).

The tenodesis site is prepared using a burr or an osteotome. The tendon is transected approximately 2.5 cm proximal to the myotendinous junction and sutured in a modified whip-stitch manner using a No.2 FiberWire, with the suture tails left at the stump (Figure 1c1). In cases of chronic tears, the retracted tendon is marked 2.5 cm proximal to the myotendinous junction and fixed under appropriate tension (26).

Depending on the tendon stump size, a 6–8 mm hollow drill is used to create the distal bone tunnel through the anterior humeral cortex. A 2.5 mm unicortical drill hole is then made 2 cm proximal to this as the proximal tunnel. A curved suture passer is introduced through the proximal hole, and its loop is retrieved from the distal tunnel (Figure 1c2). The two suture tails are passed through the loop, pulled from the distal into the proximal tunnel, and threaded through the suture button. The tendon is docked intramedullary within the distal tunnel. After achieving adequate tension, the sutures are tied and secured over the button (Figure 1c3) (26).

#### 2.3.2 Advantages and limitations

The unicortical suture button fixation technique eliminates the need for far cortex penetration, thereby effectively protecting contralateral neurovascular structures. The absence of cortical implants also reduces the risk of diaphyseal stress concentration and subsequent fractures. This technique enables direct visualization during the establishment of stable cortical-intramedullary tendon fixation (26). Regarding biomechanical performance, cadaveric studies by Ahmad et al. (27) demonstrated that suture buttons exhibit superior ultimate failure strength and greater resistance to cyclic displacement compared to interference screws. Furthermore, its intramedullary fixation approach better preserves tendon structural integrity and protects the axillary nerve (26). Consequently, the authors believe that this technique is particularly suitable for patients with compromised tendon quality, such as those with chronic biceps tendinopathy. However, it requires the use of implants, which inevitably increases the economic burden on patients.

### 2.4 Keyhole proximal biceps tenodesis

#### 2.4.1 Surgical procedure

If a subscapularis tendon tear is present, repair it first to improve exposure. Biceps tendon pathology is reserved for the final stage of the procedure (28).

The biceps tendon is transected at the supraglenoid tubercle and retracted into the bicipital groove. Three surgical portals—R, S, and A—are established. The R and S portals are positioned proximal and distal to the biceps tendon path, respectively, while the A portal is located three to four fingerbreadths distal to the anterolateral edge of the acromion (Figure 1d1). Under visualization via the A portal, a shaver or radiofrequency device is introduced through the S portal to debride soft tissue and identify the transverse humeral ligament, the bicipital groove synovium, and the superior border of the pectoralis major (28).

A percutaneous sharp incision is made in the biceps tendon sheath with an epidural needle tip. The tendon is retrieved through the S portal using a suture grasper, while limb traction is released to obtain sufficient working length (28).

Approximately 2–2.5 cm distal to the myotendinous junction, the tendon is whip-stitched and sutured with a No.2 suture to form a tendon plug. Before final suturing, a freely sliding traction suture is placed within the loop (Figure 1d2). After measuring the dimensions of the tendon plug, a K-wire with a protective sleeve is drilled into the center of the bicipital groove at a 45° angle, at the level of the superior border of the pectoralis major and the inferior edge of the transverse humeral ligament. Once the posterior cortex is penetrated, the K-wire is gently tapped until it exits the skin to minimize neurovascular injury (28).

A reamer matching the diameter of the tendon plug (typically 6–7 mm) is used over the K-wire to create a 25–30 mm deep unicortical pilot hole, with the K-wire left in place. A 5 mm offset guide is introduced through the S portal, and a second K-wire is drilled distal to the pilot hole. A 4.5 mm cannulated drill is then used to penetrate only the anterior cortex. The bone bridge between the two holes is removed, completing the “keyhole” structure (Figure 1d3) (28).

A suture loop is introduced through the R portal. Under visualization via the A portal, the traction suture is passed through the loop and retrieved posteriorly. A suture retriever is used through the S portal to advance the tendon plug toward the proximal end of the keyhole, while an assistant pulls the traction suture. An audible “click” confirms that the tendon is securely locked into the distal keyhole, after which the traction suture is removed (Figure 1d4) (28).

#### 2.4.2 Advantages and limitations

The technique is safe, easy to master, cost-effective, and requires a relatively short operative time (approximately 15–20 min). No special instruments are needed, facilitating its clinical adoption. It provides clear visualization of the entire biceps tendon and its sheath, allowing thorough evaluation for occult tears, synovitis, or fibrosis within the sheath or distal to the tendon (28). However, certain limitations remain: the technique offers limited initial stability, requires working through the interval between the pectoralis major and deltoid muscles, and may lead to postoperative pain and suboptimal cosmetic outcomes (29).

### 2.5 “Onlay” fixation technique

#### 2.5.1 Surgical procedure

The LHBT is detached from the supraglenoid tubercle. The shoulder is placed in 90° abduction and the elbow in 90° flexion (30).

**Figure 2:**
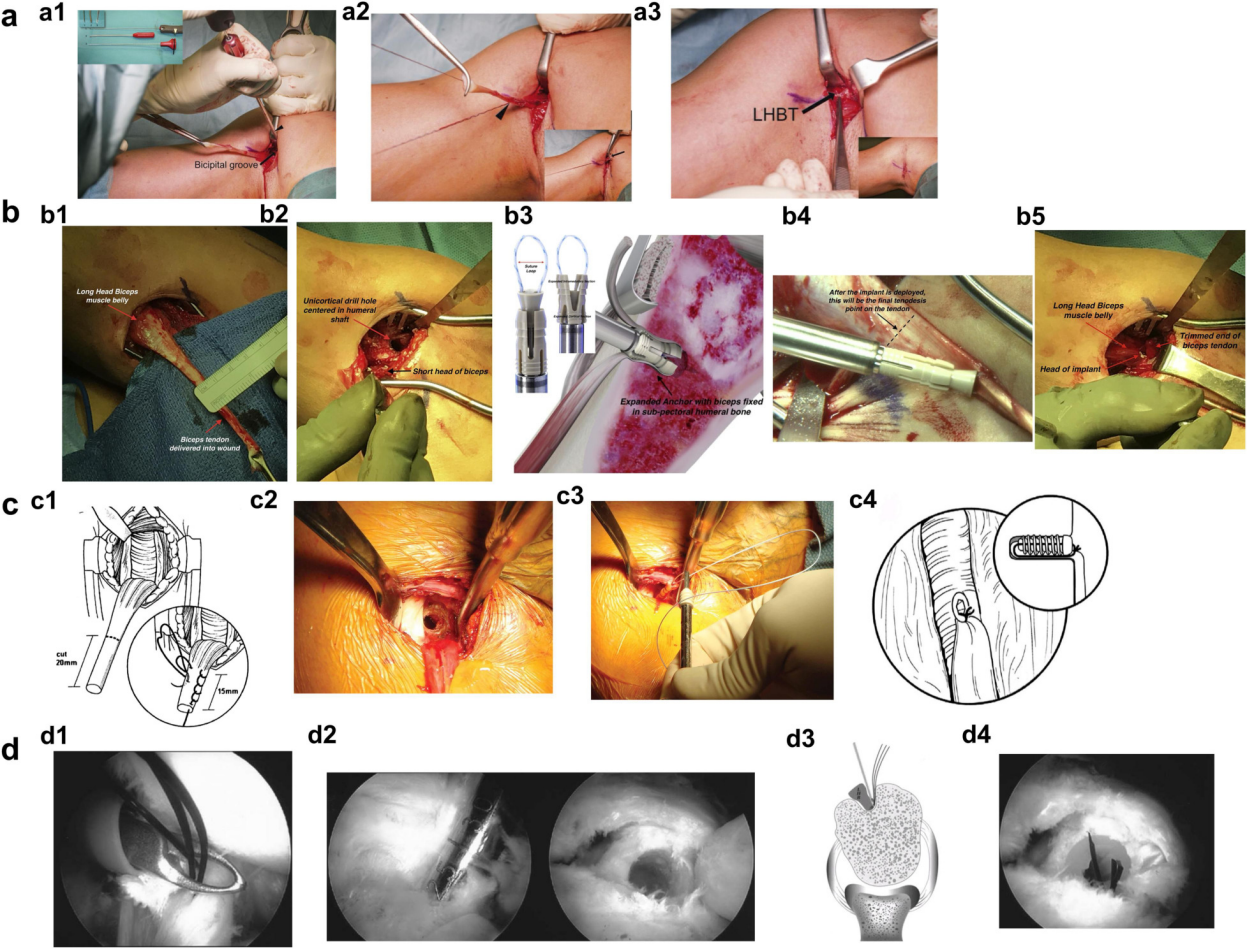
Diagram of the key steps for the “onlay” fixation technique (**a**), expandable PEEK device fixation technique (**b**), absorbable interference screw fixation technique (**c**), and end-tunnel fixation technique (**d**). (**a1**) Creation of a guided monocortical tunnel (1.6 mm) in the bicipital groove. (**a2**) A running locked suture with four passes is placed across the myotendinous junction. (**a3**) Suture fixation compressing the tendon against the anchor. Panel (**a**) is adapted from (30). (**b1**) Tendon diameter measurement for implant sizing. (**b2**) A monocortical tunnel is drilled over a centrally placed guidewire in the distal bicipital groove. (**b3**) The tendon is passed through the TenoLok anchor suture loop. (**b4**) Distal implant fixation aligned with the myotendinous junction. **(b5**) The excess tendon is excised to preserve a 1 cm proximal stump. Panel (**b**) is adapted from (32). (**c1**) Preparation of the biceps tendon, showing a 20 mm stump whip-stitched to within 15 mm of the myotendinous junction. (**c2**) Creation of a 15 mm deep bone tunnel following guide pin placement. (**c3**) After debris removal, one suture is loaded through the screw and screwdriver, leaving the other free. (**c4**) The screw is advanced into the tunnel until flush, and the sutures are tied to achieve dual fixation via interference and suture anchor principles. Panel (**c**) is adapted from (22). (**d1**) Three No.0 PDS sutures are successively passed and retrieved through the anterior portal. (**d2**) Determination of the anchor trajectory followed by creation of a deep bone tunnel. (**d3**) Sutures retrieved through the anterolateral portal and loaded onto the guide pin eyelet. (**d4**) Final tendon fixation with a bioabsorbable interference screw. Panel (**d**) is adapted from (39).

An incision is made along the inferior border of the pectoralis major. The LHBT is exposed and retrieved through the incision using a right-angle clamp. The periosteum over the bicipital groove is cleared, and a drill guide is positioned centrally within the groove, approximately 2 cm proximal to the inferior margin of the pectoralis major. A monocortical bone tunnel is created using a 1.6 mm drill bit (Figure 2a1) (30).

A single-load full-suture anchor (Fibertak) is employed. One suture is used to perform a running locked suture along the tendon, spanning from 1 cm proximal to 2 cm distal to the myotendinous junction, with a total of four passes (Figure 2a2). The remaining proximal portion of the tendon is sharply excised. The suture tails are passed through the tendon, pulled to advance and compress the tendon against the anchor, and finally tied securely (Figure 2a3) (30).

#### 2.5.2 Advantages and limitations

By releasing the proximal tendon, this technique effectively alleviates pain while simultaneously reducing the potential risk of humeral fracture. Furthermore, the surgical approach is straightforward: the use of a soft-tissue drill and guide enables precise anchor placement and facilitates access to the bicipital groove beneath the inferior border of the pectoralis major, thereby shortening operative time (30). Siebenlist et al. (31) reported favorable clinical outcomes and a low failure rate using the onlay technique for acute distal biceps tendon tears.

Nevertheless, several limitations warrant consideration. During the inferior pectoralis major approach, the interval between the pectoralis major tendon and the short head of the biceps tendon is often difficult to visualize clearly; in such cases, palpating the inferior edge of the pectoralis major tendon can assist in localization. Moreover, anchor malposition may occur during insertion; therefore, accurate identification of the bicipital groove and central placement of the guide are essential. Another potential issue is inadequate tension after tendon fixation; accordingly, suturing should extend from 1 cm proximal to 2 cm distal to the myotendinous junction to better restore native tension. Finally, there remains a potential risk of iatrogenic neurovascular injury (30).

### 2.6 Expandable PEEK device fixation technique

#### 2.6.1 Surgical procedure

After arthroscopic management of associated intra-articular lesions, the LHBT is transected. The shoulder is positioned in 20° abduction, 10° external rotation, and 0° flexion. A subpectoral incision is made, and the biceps tendon is delivered proximally (32).

Tendon diameter is measured 15 mm proximal to the myotendinous junction to determine drill and implant size (Figure 2b1). Retracting the pectoralis major laterally and the biceps short head medially exposes the distal bicipital groove. A guidewire is inserted 5 mm proximal to the inferior pectoralis major border, centered in the humeral shaft to reduce fracture risk. A monocortical tunnel is drilled over the guidewire (Figure 2b2) (32).

The tendon is passed through the TenoLok anchor suture loop (Figure 2b3) and tensioned 17 mm proximal to the myotendinous junction. The implant is secured distally to the tendon, with its head aligned to the myotendinous junction (Figure 2b4). The anchor-tendon construct is tapped into the tunnel until flush with the cortex (32).

The device is expanded by stabilizing the white component and rotating the black knob until a distinct click confirms full expansion. After ensuring the suture loop is untangled, the handle and guide are removed. A 1 cm proximal tendon stump is preserved, and excess tendon excised (Figure 2b5) (32).

#### 2.6.2 Advantages and limitations

The device features a single-suture loop design. With a pre-insertion diameter of 5–6 mm, it expands radially after implantation to conform to the humeral medullary cavity. Once the expansion screw is deployed, both its head and base contribute to a combined intramedullary-cortical fixation. This mechanism eliminates the need for suture penetration through the tendon, thereby avoiding additional iatrogenic tendon damage. Compared with threaded devices, this design is particularly suitable for the densely bony pectoral region and reduces potential tendon injury. A suture loop at the device base helps maintain the anatomical position and length of the biceps tendon during implantation. The technique is reliable and reproducible, and the procedure can be completed within 15 min once proficiency is gained (32).

However, the technique has several limitations: it requires an additional incision with associated risks of injuring the musculocutaneous nerve or cephalic vein; creating a 6–8 mm bone tunnel increases the risk of humeral fracture or, if the far cortex is breached, potential injury to the axillary nerve; and the specialized implant adds extra cost (32).

### 2.7 Absorbable interference screw fixation technique

#### 2.7.1 Surgical procedure

The LHBT is transected near its insertion. An incision is made to expose the inferior border of the pectoralis major. The fascia overlying the coracobrachialis and biceps muscles is incised in a proximal-to-distal direction. The pectoralis major is retracted proximally and laterally with a Hohmann retractor, while the coracobrachialis and short head of the biceps are retracted medially (22).

The biceps tendon is delivered through the incision from the deep layer using a right-angle clamp. It is transected 20 mm from its proximal end, and the tendon is whip-stitched up to 15 mm proximal to the myotendinous junction (Figure 2c1) (22).

A guide pin is inserted at the center of the inferior border of the pectoralis major, 1 cm proximal to the bicipital groove. An 8 mm drill is used to create a 15 mm deep bone tunnel (Figure 2c2). After bone debris is removed, one suture is passed through the screw (8  ×  12 mm) and screwdriver, while the other suture is left free (Figure 2c3) (22).

The screwdriver is inserted into the bone tunnel, and the screw is advanced over the tendon until it is flush with the tunnel entrance. The screwdriver is then removed. The suture through the screw is tied to the free suture, achieving a dual fixation construct: interference compression by the screw and suture anchor fixation via the tied sutures (Figure 2c4) (22).

#### 2.7.2 Advantages and limitations

The absorbable interference screw fixation technique offers distinct advantages, beginning with its superior biomechanical performance demonstrated through the highest mean ultimate failure load among available techniques, ensuring exceptional initial fixation strength and stability (31). Clinically, it maintains a low complication profile with only 2% overall incidence while achieving significantly reduced persistent bicipital groove tenderness (3% vs. 7% with suture anchors) (33, 34). Furthermore, clinical evidence confirms substantial symptom improvement, as shown in a 50-patient cohort where all postoperative outcome measures improved significantly, validating the technique's effectiveness for symptomatic biceps tendon pathology (10).

However, several limitations warrant consideration, including the persistent pain in the biceps tendon and potential for fixation failure that may lead to “Popeye” deformity (34). The procedure also carries implant-specific risks such as foreign body reactions to bioabsorbable materials. Most notably, fracture risk increases due to stress concentration effects from bone tunnel creation; malpositioned screws can reduce humeral strength by 25% (35), with documented clinical cases highlighting this vulnerability (19, 36).

### 2.8 Summary and comparison of the above seven subpectoral tenodesis techniques

Based on the details and respective strengths and limitations of the seven techniques mentioned above, we compared them across five key aspects: implant requirement, risk of iatrogenic biological injury, risk of iatrogenic fracture, operative difficulty, and fixation strength. The results of this comparison are summarized in Table 1.

**Table 1:** Comparison of seven subpectoral tenodesis techniques.

Technique name	Implant required	Risk of iatrogenic nerve injury	Risk of iatrogenic fracture	Operative difficulty	Fixation strength (theoretical comparison)
Three-tunnel fixation	No	Medium	Medium	Medium (requires precise creation and matching of three tunnels)	Medium (relies on surgeon's skill and the quality of the bone tunnels)
Double-tunnel fixation	No	Medium	Medium	Low	Medium
Unicortical suture button fixation	Yes (Suture button)	Low	Low	Medium	High (superior ultimate failure load and resistance to displacement)
Keyhole proximal biceps tenodesis	No	Low	Low	Low-Medium	Medium (limited initial stability)
"Onlay” fixation	Yes (Suture anchor)	Medium (potential risk of neurovascular injury)	Low	Medium (requires accurate identification of bicipital groove and anchor placement)	Medium
Expandable PEEK device fixation	Yes (Expandable PEEK implant)	Medium	Medium	Medium	Medium (Combined intramedullary-cortical fixation)
Absorbable interference screw fixation	Yes (Absorbable interference screw)	Low	High (stress concentration from drilling; malpositioned screws can reduce humeral strength by up to 25%)	Medium (involves precise drilling and screw placement)	High (the highest mean ultimate failure load)

Overall, the authors believe that there is no single “all-purpose” technique. The choice of subpectoral tenodesis technique requires a comprehensive balance between the patient's tendon condition, fixation strength, safety, procedural complexity, and economic considerations.

## 3 Non-subpectoral tenodesis

Non-subpectoral tenodesis is typically performed arthroscopically. During the procedure, diagnostic arthroscopy is conducted, and the LHBT is fixed in the region between the top of the bicipital groove and the superior border of the pectoralis major, while associated injuries can be addressed concurrently. These techniques offer the advantage of being minimally invasive, requiring no additional incision, and are associated with relatively lower risks of fracture and nerve injury. Research by Boileau and Neyton demonstrated that post-operative biceps strength on the operated side can reach 90% of the contralateral side (37). However, these techniques also have certain limitations: arthroscopic execution is technically demanding with a steep learning curve; improper suture management during the procedure can lead to confusion of suture tails, potentially prolonging operative time; furthermore, due to the relatively proximal fixation site, it may not allow for thorough removal of the biceps tendon sheath and associated synovial tissue within the bicipital groove, potentially resulting in residual anterior shoulder pain. An *in vitro* study also suggests that the suprapectoral fixation technique may have a lower failure load compared to the subpectoral approach (38).

### 3.1 End-tunnel fixation technique

#### 3.1.1 Surgical procedure

A spinal needle is introduced percutaneously through the rotator cuff interval near the anterolateral acromion to penetrate and mark the LHBT at its proximal tenotomy site. A No.0 PDS suture is passed through the needle, grasped with a suture retriever, and the needle is withdrawn. The opposite end of the suture is pulled out through the anterior portal, and both tails are secured with a hemostat. This process is repeated to place three PDS sutures (Figure 2d1). After addressing concomitant intra-articular pathologies, the biceps tendon is transected at its origin on the supraglenoid tubercle. The bicipital groove is exposed, and the tenodesis site is identified just distal to the articular cartilage margin. A spinal needle is used to determine the correct trajectory for anchor placement, followed by creation of a bone tunnel using a cannulated drill, 6–7.5 mm in diameter and 25 mm deep (Figure 2d2). All sutures are retrieved through the anterolateral portal and threaded through the eyelet of a 2.0 mm guide pin. The biceps tendon is shuttled into the bone tunnel using the pin (Figure 2d3). Once the pin contacts the tunnel base, a bioabsorbable interference screw is inserted. The pin is removed after the screw is more than halfway advanced, and the screw is fully seated to complete fixation (Figure 2d4) (39).

#### 3.1.2 Advantages and limitations

This technique is relatively simple and can be performed using standard instrumentation. The sequential dilation of the humeral bone tunnel may enhance fixation pullout strength, and the procedure carries no risk of major neurovascular injury (39). However, the authors believe that the use of interference screws increases the risk of iatrogenic fractures, and the fixation strength requires further validation and investigation.

### 3.2 Knotless suture fixation technique

#### 3.2.1 Surgical procedure

If the bicipital groove and the transverse humeral ligament have not been exposed in the subacromial space, a suture marker may be placed during glenohumeral arthroscopy to localize the LHBT. The intra-articular portion of the LHBT is left attached until the tenodesis is completed, which facilitates manipulation with a suture grasper (40).

**Figure 3:**
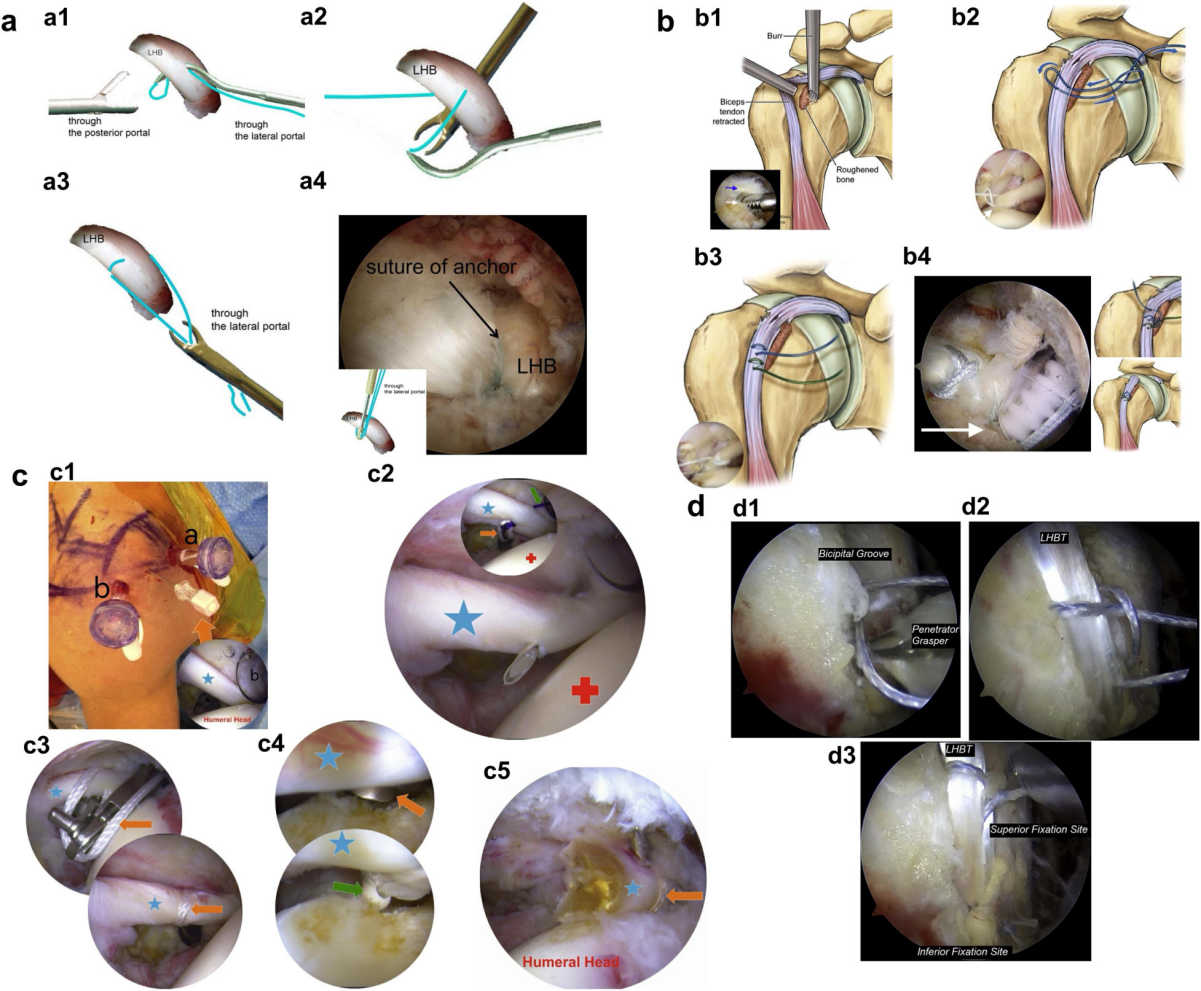
Diagram of the key steps for the knotless suture fixation technique (**a**), arthroscopic onlay articular margin suture anchor fixation technique (**b**), suprapectoral lasso-loop knotless fixation technique (**c**), and double lasso-loop fixation technique (**d**). (**a1**) A No.2 non-absorbable suture is passed through a suture hook, which is then used to penetrate the LHBT at the bicipital groove. (**a2**) The external suture tail is retrieved posteriorly, and the internal tail is grasped anteriorly by flipping the suture hook. (**a3**) Both suture limbs are retrieved together through the lateral portal cannula. (**a4**) Final fixation with a knotless suture anchor after tensioning and trimming the sutures. Panel (**a**) is adapted from (40). (**b1**) Preparation of a tendon fixation bed at the bicipital groove apex using a burr. (**b2**) A looped No.2 FiberWire is passed twice through the upper third of the LHBT and secured by retrieving the tail through the loop. (**b3**) A second suture passage is placed adjacent to the first. (**b4**) Final reinforced fixation with a SwiveLock anchor and additional suture passage and tying. Panel (**b**) is adapted from (43). (**c1**) Portal placement within the rotator interval following confirmation of biceps pathology. (**c2**) A No.1 PDS suture is passed through the biceps tendon and used to shuttle a suture tape. (**c3**) Completion of the lasso-loop configuration around the biceps tendon using suture tape. (**c4**) Anchor fixation of the lasso-loop suture. (**c5**) Final assessment of tenodesis stability following stump ablation. Panel (**c**) is adapted from (44). (**d1**) Secure fixation achieved with a FiberTak suture anchor placed inferior to the bicipital groove. (**d2**) A lasso-loop suture is configured around the biceps tendon. (**d3**) Dual lasso-loop fixation of the biceps tendon with two suture anchors. Panel (**d**) is adapted from (45).

A No.2 non-absorbable suture is first passed through the lumen of a suture hook. The loaded suture hook is introduced through the lateral portal cannula and penetrates the mid-portion of the LHBT at the bicipital groove (Figure 3a1). A suture grasper is inserted through the posterior portal to retrieve the external tail of the suture hook and pull it out. Another grasper is then introduced through the anterior portal, passes beneath the LHBT, and flips the suture hook into its jaws to grasp the internal tail of the suture, which is then pulled out through the anterior portal (Figure 3a2) (40).

Both the anterior and posterior limbs of the suture are retrieved together through the lateral portal cannula (Figure 3a3). The cortex of the bicipital groove is decorticated using a drill, and a bone tunnel is created with an awl. The suture ends are passed through the eyelet of a knotless suture anchor (Bio-PushLock). Under appropriate tension, the anchor is tapped into the bone tunnel. The suture tension is verified, and excess suture tails are trimmed (Figure 3a4). Finally, the intra-articular portion of the LHBT is transected (40).

#### 3.2.2 Advantages and limitations

This technique offers several advantages. First, it can be performed using conventional instruments without the need for specialized equipment. Second, compared to suture anchor techniques, which typically fix only a portion of the tendon thickness, this method encircles the entire tendon circumference, theoretically enhancing fixation strength. Additionally, the risk of suture slippage during tendon penetration is low. In a series of 30 biceps tenodesis procedures performed using this technique, short-term follow-up revealed only one case of Popeye deformity (40).

However, the technique also has certain limitations. It achieves only point contact between the tendon and bone, whereas interference screw techniques provide a larger contact area. Patzer et al. (41) reported significantly higher load-to-failure with interference screws compared to knotless suture anchors in a human cadaveric study. In contrast, other laboratory studies have presented conflicting data (42). Notably, Millett and colleagues (33) found no significant differences in clinical outcomes between interference screw and suture anchor techniques. If the aim is to emphasize procedural simplicity and safety (particularly when bone quality is a concern), the authors believe that the knotless suture fixation technique represents a reasonable choice.

### 3.3 Arthroscopic onlay articular margin suture anchor fixation technique

#### 3.3.1 Surgical procedure

The rotator interval is fully opened, with partial coracoacromial ligament resection if necessary. A cannula is introduced through the anterior portal. The bicipital groove is identified along the superior aspect of the LHBT, and the transverse humeral ligament is released from superior to inferior, followed by dislocation of the LHBT (43).

A tendon fixation bed is prepared at the apex of the bicipital groove using a burr with a protective sleeve (Figure 3b1). A suture passer is then used to pass a looped No.2 FiberWire through the upper third of the LHBT at the groove apex. The suture is passed twice through the tendon, and the tail is retrieved through the loop (Figure 3b2). The same procedure is repeated adjacent to the first suture passage (Figure 3b3) (43).

The two suture tails are threaded through a Biocomposite Double-Loaded SwiveLock anchor. After pilot hole preparation, the anchor is inserted under appropriate LHBT tension until the flange is flush with the cortical bone. One tail is then passed through the tendon again, and both tails are tied to reinforce the fixation (Figure 3b4) (43).

Finally, the LHBT is transected at the supraglenoid tubercle, and residual tendon tissue is debrided. In cases accompanied by rotator cuff tear, a standard lateral portal may be established through the tear for groove management, LHBT passage, and anchor insertion (43).

#### 3.3.2 Advantages and limitations

The key advantages of this technique include: (1) direct visualization for confirming appropriate length-tension relationship of the tendon, ensuring the distal biceps remains stable without retraction after release from the supraglenoid tubercle; (2) requirement of only standard arthroscopic portals without additional incisions; and (3) anchor placement in the high-density bone at the glenoid rim, providing reliable fixation (43).

Limitations include the risk of postoperative tendon rupture; in case of fixation failure, revision tenodesis below the pectoralis major may be required. Additionally, adequate rotator interval release is necessary to ensure sufficient working space for instrumentation (43).

### 3.4 Suprapectoral lasso-loop knotless fixation technique

#### 3.4.1 Surgical procedure

Following diagnostic arthroscopy and confirmation of biceps tendon pathology, two portals are established using spinal needle localization: an anterior portal (a) within the rotator interval, and an anterosuperolateral portal (b) positioned superiorly within the rotator interval adjacent to the anterior edge of the supraspinatus tendon. Two 6.0-mm threaded cannulas are then inserted (Figure 3c1) (44).

A spinal needle is used to perforate the biceps tendon and pass a No.1 PDS suture, which is retrieved through portal a. The other end of the PDS suture is grasped and pulled out through portal b. Extracorporeally, the proximal end of the PDS suture is knotted to assist in shuttling a suture tape through the biceps tendon, while the distal end is tied to the tape to facilitate its passage (Figure 3c2) (44).

A suture grasper is introduced through portal b into the glenohumeral joint to grasp and pull the superior tail of the suture. A tape grasper is then inserted through portal a, captures the tape superior to the tendon, and wraps it around the anterior aspect of the biceps to form a loop. With the grasper holding the loop, it is used to retrieve the inferior tail of the tape beneath the tendon through portal b. Both ends of the tape are then pulled out through portal a, completing the lasso-loop configuration (Figure 3c3) (44).

To optimize the length-tension relationship, the biceps tendon is released from its superior glenoid attachment. The two tails of the lasso-loop suture are retrieved through portal a (alternatively, portal b may be used for tenodesis) and threaded through a 2.9-mm PushLock anchor. A hole is drilled at the top of the bicipital groove, the sutures are tensioned, and the anchor is advanced into the bone tunnel (Figure 3c4). The tendon stump is ablated with radiofrequency, and fixation stability is assessed with an arthroscopic probe (Figure 3c5) (44).

#### 3.4.2 Advantages and limitations

This technique provides a simplified all-intra-articular fixation approach that avoids the use of an interference screw, thereby reducing the risk of postoperative anterior shoulder pain from overtensioning the biceps tendon, as well as eliminating the potential for humeral fracture associated with larger-diameter screws (19). A 2.9 mm PushLock anchor is employed for fixation, which minimizes fracture risk while omitting the need for knot tying, thus simplifying the arthroscopic procedure and reducing operative time. Moreover, the present technique utilizes a lasso-loop configuration with a 1.5 mm suture tape, which increases the contact area with the tendon and may enhance fixation strength (44).

Several limitations exist: establishing portal b along the anterior supraspinatus tendon can be challenging in patients with an intact rotator cuff; initial suture management using PDS and tape may be technically demanding; and in the beach-chair position, optimal drill positioning for the PushLock anchor may be obstructed by the patient's head (44).

### 3.5 Double lasso-loop fixation technique

#### 3.5.1 Surgical procedure

The patient is placed in the beach-chair position, with the affected shoulder flexed at 45°, abducted at 30°, and externally rotated between 10° and 20°. A standard posterior portal is used to inspect the intra-articular portion of the LHBT, evaluating the extent of tearing and the condition of the tendon sheath. The LHBT is then transected via an anterior portal, and any residual soft tissue stump at the insertion site is debrided (45).

Following subacromial bursectomy, the anterior aspect of the humeral head is visualized through a lateral portal to identify the residual LHBT. The bicipital groove is confirmed using a probe. The LHBT is tensioned to verify its anatomical position, then released within the groove (45).

A 2.6 mm FiberTak suture anchor is placed inferior to the bicipital groove, and the sutures are tensioned to achieve secure fixation (Figure 3d1). Through an anterior portal, one suture tail is passed through the mid-substance of the biceps tendon. The other tail is then retrieved anterior to the tendon to form a loop, through which the same tail is regrasped to create a lasso configuration (Figure 3d2). The two suture tails are tied to complete the first lasso-loop fixation. A second anchor was inserted 3–5 cm above the first anchor, and the same technique was repeated to accomplish the second lasso-loop fixation (Figure 3d3) (45).

#### 3.5.2 Advantages and limitations

This technique utilizes double anchor fixation, which enhances biomechanical strength by distributing stress compared to the single anchor method. The use of 2.6 mm diameter anchors helps reduce the risk of humeral fracture. In contrast to open subpectoral fixation techniques, this approach decreases the risk of iatrogenic brachial plexus injury, requires no additional incision, and minimizes scar formation. At the 6-month postoperative follow-up, it demonstrated a lower failure rate than single anchor fixation (45).

However, the technique also has certain limitations: the use of two anchors increases both surgical costs and operation time, posing greater challenges for beginners. Additionally, improper tensioning during biceps tenodesis may lead to persistent postoperative pain or joint stiffness (45).

### 3.6 360° double lasso-loop fixation technique

#### 3.6.1 Surgical procedure

First, the intra-articular biceps tendon is assessed, and the footprint of the rotator cuff is freshened. A suture anchor is placed at the proximal humeral head near the articular cartilage margin. One suture limb is used to fix the biceps tendon, while the other limb is either retrieved through a posterior portal or left outside the cannula for potential repair of the anterior supraspinatus tendon (46).

**Figure 4:**
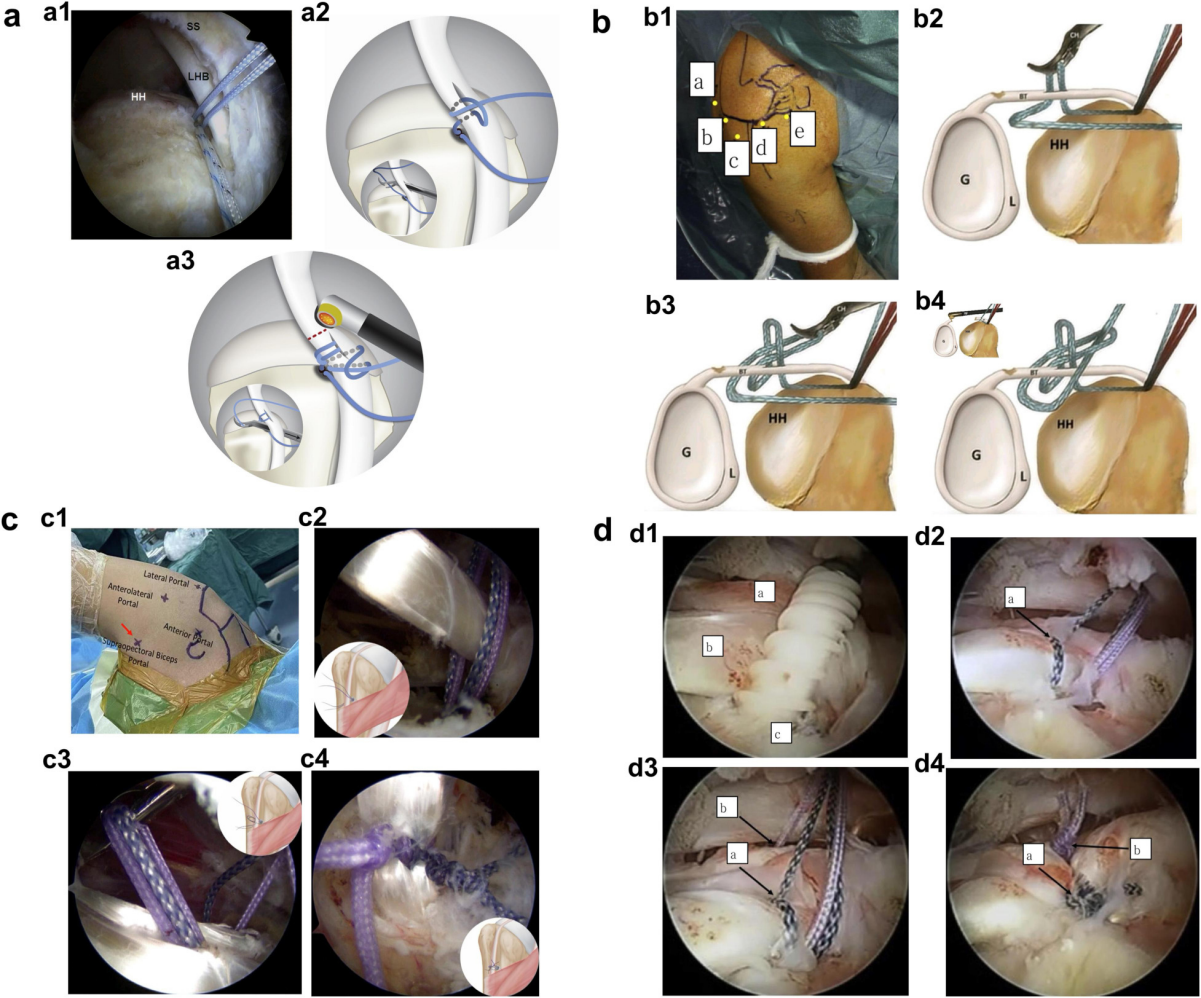
Diagram of the key steps for the 360° double lasso-loop fixation technique (**a**), loop-within-a-loop technique (**b**), double 360° double lasso-loop fixation technique (**c**), and “sandwich” tenodesis technique (**d**). (**a1**) suture passage through the biceps tendon. (**a2**) Creation of the first lasso loop above the biceps tendon. (**a3**) Completion of the second lasso loop anterior to the biceps tendon. Panel a is adapted from (46). (**b1**) Arthroscopic portal placement. (**b2**) Formation of the first lasso loop using a supinated suture passer. (**b3**) The same suture is regrasped through the first loop to create the second lasso loop. (**b4**) The suture construct is completed by retrieving the free posterior limb through the second loop. Panel (**b**) is adapted from (49). (**c1**) Arthroscopic portal location (right shoulder): the suprapectoral biceps portal is indicated by the red arrow at the axillary fold apex. (**c2**) A double-suture anchor is inserted posteriorly within the bicipital groove, positioning the sutures on either side of the LHBT. (**c3**) A suture passer is advanced through the biceps tendon to retrieve one strand from each of the two differently colored sutures, forming two loops upon withdrawal. (**c4**) Completion of two lasso loops around the biceps tendon by passing the free limbs through their corresponding loops. Panel (**c**) is adapted from (50). (**d1**) Insertion of a double-suture anchor. Labeled structures: (a) rotator cuff interval, (a) long biceps tendon, (c) hole in bicipital groove. (**d2**) Creating a lasso loop (black arrow) around the biceps tendon with a blue suture. (**d3**) Placement of a purple suture (b, black arrow) beneath the LHBT and through the rotator cuff interval, positioning it next to the existing lasso loop (a, black arrow). (**d4**) Final construct after tying the sutures, showing (a) purple knot (tendon-to-tendon fixation) and (b) blue knot (tendon-to-bone fixation). The blue sutures were tied prior to the purple. Panel (**d**) is adapted from (52).

Using the lateral portal as the viewing portal, a suture retriever is introduced through the anterolateral portal to place one end of the suture into the glenohumeral joint. A suture hook is then passed through the center of the biceps tendon to grasp this suture limb (Figure 4a1). After withdrawing the hook, a loop is formed above the tendon. The suture retriever is passed through this loop to grasp the same suture limb again, creating the first lasso loop (Figure 4a2). A second loop is then created by grasping the same limb anterior to the biceps tendon, forming the second lasso loop (Figure 4a3). Finally, the suture is tightened and secured with knots (46).

#### 3.6.2 Advantages and limitations

This technique is safe, reproducible, versatile, and cost-effective. It features straightforward steps and excellent intraoperative visualization, facilitating both teaching and adoption. The procedure carries a low risk of neurovascular injury or humeral fracture, requires a short operative time (typically under five minutes), and obviates the need for specific anchors or implants. Sutures from the same anchor can also be used to address concomitant pathologies involving the anterior margin of the supraspinatus tendon (46). Biomechanical studies indicate that self-cinching sutures provide superior fixation strength at the tissue-suture interface compared with non-self-cinching sutures (47). Other research has shown that modified lasso-loop sutures yield tenodesis results equivalent to those achieved with interfacial screws (48).

The potential limitations of this technique are similar to other biceps tenodesis procedures performed high in the intertubercular groove. If persistent anterior shoulder pain due to residual tendinopathy is a concern, fixation may be performed within or below the groove rather than adjacent to the articular margin. Another possible issue is residual biceps tissue covering the rotator cuff footprint, which may interfere with healing. Meticulous removal of excess tendon around the fixation site is therefore recommended to avoid this complication (46).

### 3.7 Double 360° double lasso-loop fixation technique (loop-within-a-loop technique)

#### 3.7.1 Surgical technique

A posterior portal is established to enter the glenohumeral joint and assess the biceps tendon and rotator cuff tear. The viewing portal is selected as either the posterior portal (a) or the lateral portal (c), which forms an equilateral triangle with portals b and d. An anterior portal (e) is established for debridement (Figure 4b1). The rotator cuff interval is sufficiently released (49).

The portal strategy is adjusted according to the tear size: for small tears, an anterolateral portal is created at the anterior margin of the supraspinatus tendon, with a posterolateral portal added as a working/accessory portal if needed. For extensive posterosuperior tears, the lateral portal is used for viewing, and bursal tissue around it is cleared (49).

A suture anchor is placed posterosuperiorly in the bicipital groove to secure the biceps tendon. The residual suture limbs can be used to repair small anterior supraspinatus tears. Positioning the anchor posterior to the bicipital groove facilitates suture management. A posterolateral portal is then created to retrieve sutures from the anchor and complete the first 360° lasso loop (49).

In cases with large posterosuperior tears, viewing from the lateral portal, three strands of the anchor sutures are retrieved via the posterior/posterolateral portals, while the remaining strands are left in the anterior portal. Radiofrequency marking and partial tenotomy of the biceps tendon help identify the accurate suture passage site (49).

The construction of the lasso loops involves the following key steps:

(1) Via the lateral/anterolateral portal, a suture is passed from the posteroinferior aspect of the biceps tendon and advanced medially along the superior border of the subscapularis tendon.
(2) Through the anterior portal, a penetrating suture passer is introduced in a supinated manner (clockwise in right shoulders, counterclockwise in left shoulders) to penetrate the tendon and retrieve the suture, forming the first lasso loop (Figure 4b2).
(3) The same suture is regrasped from its posteromedial segment through another tendon penetration and pulled through the first loop to create the second lasso loop (Figure 4b3).
(4) Finally, the free limb posterior to the biceps tendon is grasped through the second loop and withdrawn through the anterolateral portal (Figure 4b4).

After identifying the post limb, a knot marker is placed. Both loops are sequentially tightened and secured, followed by resection of the distal biceps tendon (49).

#### 3.7.2 Advantages and limitations

This technique is a reproducible arthroscopic tenodesis method. Its relatively straightforward procedure effectively neutralizes opposing traction forces through tendon-to-tendon suturing. Furthermore, it also allows for the use of the same implants as those employed in rotator cuff repair, contributing to reduced surgical costs. In addition, the technique demonstrates clear advantages in preventing “Popeye” deformity and improving cosmetic outcomes (49).

However, several limitations should be noted: intraoperative care must be taken to avoid suture confusion risk, only non-sliding knots can be used for fixation, and the procedure is not feasible when the LHBT is scarred and immobile within the intertubercular groove (49).

### 3.8 Double 360° double lasso-loop fixation technique

#### 3.8.1 Surgical procedure

The procedure begins with a posterior viewing portal to assess biceps tendon degeneration and injury. If tenodesis is indicated, the arthroscope is redirected to the subacromial space. An anterolateral portal is established for subacromial debridement and exposure, with acromioplasty if needed (50).

A lateral portal is then created, and the arthroscope is transferred to this portal to visualize the distal and anterior bicipital groove. The biceps tendon is fully exposed from the proximal transverse humeral ligament to the distal superior border of the pectoralis major (50).

A suprapectoral biceps portal is established at the apex of the posterior axillary fold (Figure 4c1). Arthroscopic visualization confirms that the localization needle is positioned directly at the junction of the superior pectoralis major border and the biceps tendon. Through this portal, a double-suture anchor is inserted posteriorly within the bicipital groove at the superior pectoralis major border, with the two suture limbs positioned on either side of the LHBT (Figure 4c2) (50).

A penetrating suture passer is introduced through the Supra-B portal to pierce the biceps tendon proximal to the anchor site, thereby avoiding excessive tension after fixation. The biceps tendon may be lifted via an anterior portal using a suture grasper to facilitate penetration (50).

The tip of the penetrating suture passer is advanced through the mid-substance of the biceps tendon to sequentially retrieve one strand from each of the two differently colored sutures located on either side of the tendon. Upon withdrawal, two loops are formed (Figure 4c3). The free limbs of the loops anterior and posterior to the biceps tendon are then retrieved through the anterior and posterior portals, respectively, passing through the corresponding loops to construct two lasso loops around the tendon (Figure 4c4) (50).

Finally, all suture limbs are tightened and secured with knots. The stability and appropriate tension of the biceps tenodesis are confirmed. The biceps tendon is transected at the supraglenoid tubercle, and the residual stump is managed (50).

#### 3.8.2 Advantages and limitations

This technique employs a four-strand double-suture anchor for biceps tenodesis, characterized by its procedural simplicity and short learning curve. A key advantage is the ability to construct bilateral 360° lasso loops through a single tendon penetration, which minimizes iatrogenic tendon damage and helps preserve the structural integrity of the fixation. The bilateral loop configuration enables balanced force neutralization upon knot tying, thereby enhancing overall stability and fixation strength (50). Since the initial description of the single lasso-loop technique by Lafosse et al. (51) in 2006, various double lasso-loop modifications have been developed, demonstrating that such techniques are straightforward, reproducible, and provide robust fixation, earning broad acceptance in shoulder surgery (45, 49). The novel double 360° lasso-loop fixation technique preserves these advantages while further reducing iatrogenic tendon injury. However, additional clinical and biomechanical studies are needed to evaluate the long-term reliability of this tenodesis method (50).

### 3.9 The “sandwich” tenodesis technique

#### 3.9.1 Surgical procedure

After standard portal establishment and synovectomy, the bony bed is prepared at the proximal aspect of the bicipital groove. The groove is debrided and exposed down to bleeding subchondral bone to create an optimal healing interface (52).

A double-suture anchor is inserted (Figure 4d1). Four color-coded sutures are managed separately. First, a blue suture is passed through the biceps tendon using a penetrating grasper and looped in a lasso configuration (Figure 4d2). Next, a purple suture is placed beneath the LHBT and retrieved through the rotator cuff interval, positioning sutures on either side of the tendon (Figure 4d3) (52).

The two blue sutures are tied first for tendon-to-bone fixation, followed by the two purple sutures for tendon-to-tendon fixation (Figure 4d4). This “sandwich” configuration enhances the stability of conventional tendon-to-bone fixation alone and is expected to promote healing (52).

#### 3.9.2 Advantages and limitations

This technique offers a straightforward all-arthroscopic tenodesis solution that combines both bony and soft tissue fixation. The procedure involves first fixing the LHBT in the bicipital groove, followed by reinforcement using the rotator cuff interval tissue (52). This design may offer biological advantages, as it promotes healing between two similar types of tissues—previous studies have shown that healing between homogeneous tissues yields better outcomes compared to heterogeneous tissues (53–55). The technique can be performed either arthroscopically or via an open approach. According to the researchers, groove positioning is faster and technically less demanding, with no increase in complication rates (52).

The main limitations include a learning curve associated with the procedure and the requirement for a small amount of additional time to establish the second fixation point (52).

### 3.10 Summary and comparison of the above nine non-subpectoral tenodesis techniques

Based on the details and respective strengths and limitations of the nine techniques mentioned above, we compared them across five key aspects: implant requirement, risk of iatrogenic biological injury, risk of iatrogenic fracture, operative difficulty, and fixation strength. The results of this comparison are summarized in Table 2.

**Table 2:** Comparison of nine non-subpectoral tenodesis techniques.

Technique name	Implant required	Risk of iatrogenic nerve injury	Risk of iatrogenic fracture	Operative difficulty	Fixation strength (theoretical comparison)
End-tunnel fixation	Yes (interference screw)	Low	Medium	Low	Medium
Knotless suture fixation	Yes (suture anchor)	Low	Low	Low	Medium (encircles the entire tendon circumference, theoretically enhancing fixation strength)
Arthroscopic onlay articular margin suture anchor fixation	Yes (suture anchor)	Low	Low	Medium	High (anchor placement in the high-density bone at the glenoid rim, providing reliable fixation)
Suprapectoral lasso-loop knotless fixation	Yes (PushLock anchor)	Low	Low	Medium	High (lasso-loop configuration increases the contact area)
Double lasso-loop fixation technique	Yes (suture anchor)	Low	Low	Medium	Medium (Double anchor fixation enhances biomechanical strength)
360° double lasso-loop fixation technique	Yes (suture anchor)	Low	Low	Low	High (Comparable to interference screw)
Double 360° double lasso-loop fixation (loop-within-a-loop) technique	Yes (suture anchor)	Low	Low	Low	Medium
Double 360° double lasso-loop fixation technique	Yes (suture anchor)	Low	Low	Low	High (Requires research validation)
"Sandwich” tenodesis technique	Yes (suture anchor)	Low	Low	Medium	Medium

As we can see, the development of non-subpectoral tenodesis techniques demonstrates a clear trend toward enhancing fixation strength while continually striving for lower surgical risk, reduced trauma, and improved cost-effectiveness. In the future, more high-quality long-term comparative clinical studies will help more precisely define the optimal indications for each technique.

## 4 Discussion and prospects

Since its first introduction in 1925 (9), the LHBT tenodesis has been fundamentally aimed at preserving the optimal length-tension relationship of the biceps muscle to reduce postoperative atrophy and deformity (22, 56). The key surgical principle involves resecting the diseased tendon segment within the intertubercular sulcus while preserving overall biceps tension. Maintaining appropriate tension is critical: insufficient tension may lead to muscle deformity, fatigue, or spasm, whereas excessive tension can result in fixation failure or persistent muscle pain. Thus, precise tension control helps restore the natural contour of the biceps, preserve muscle strength, and minimize the risk of tenodesis failure (57, 58).

Regarding the choice of surgical approach and fixation location, current evidence indicates no significant differences in short- to mid-term clinical and functional outcomes between arthroscopic suprapectoral and open subpectoral biceps tenodesis (59–61). Multiple comparative studies (60, 62–66) have demonstrated that both techniques yield comparable results in postoperative shoulder range of motion, strength recovery, pain relief, and outcome scores such as ASES, with generally high patient satisfaction. Biomechanical analysis (16, 64) further confirms similar performance in terms of ultimate failure load and displacement. Even in patients with large or massive rotator cuff tears, both techniques provide equivalent improvement (65). Therefore, the choice of technique can be individualized based on specific clinical conditions and surgical considerations.

Once the surgical approach is determined, attention turns to the selection of a fixation method. A systematic review and meta-analysis (16) comparing different fixation techniques (interference screw, cortical button, suture anchor, etc.) found that interference screw fixation provided significantly greater stiffness, although no significant differences were observed in ultimate failure load or cyclic displacement. Furthermore, no significant differences in biomechanical properties were detected between suprapectoral and subpectoral fixation locations. Notably, Mazzocca et al. (23) also reported no statistically significant differences in ultimate failure load among four commonly used fixation techniques, further supporting the absence of a distinct biomechanical advantage for any single method.

While various fixation techniques demonstrate generally comparable biomechanical performance, they differ in clinical characteristics and complication risks. For interference screws, one study (67) suggested superior performance in ultimate failure load and gap formation, whereas another (68) reported an increased risk of gap formation. Additionally, complications such as implant failure, bioabsorbable screw reactions, and humeral fracture have been documented (19, 34, 35). Subpectoral tenodesis may increase the risk of stress risers due to cortical defects from drilling—a particular concern in overhead athletes (30). Although screw diameter selection shows no clear clinical advantage, larger diameters may elevate fracture risk; the management of the residual tendon may also impact postoperative pain and functional recovery (19, 44).

In specific clinical scenarios, the subpectoral biceps tenodesis demonstrates distinct value. It has been established as a safe and effective solution for revision cases following failed primary procedures. The study by Euler et al. (69) reported significant improvements in shoulder outcome scores after both primary repair of chronic tears and revision repair, with no significant differences in outcomes between the two groups. These findings are supported by other studies, which indicate that revision tenodesis effectively relieves pain, improves function, and resolves the “Popeye” deformity through complete removal of the diseased tendon from the bicipital groove (70–72).

Current evidence indicates that biceps tenodesis yields favorable outcomes across various age groups. Osbahr et al. (73) recommended the procedure for patients under 50 years of age with concomitant shoulder pathologies, such as rotator cuff tears, SLAP lesions, pain, instability, or tenosynovitis. More recent studies further support its application in younger populations (74, 75). The analysis by Eoghan et al. indicates that for symptomatic SLAP lesion patients under the age of 30, biceps tenodesis represents the most advantageous and cost-effective treatment option (74). In a study involving patients under 25 years old (75), significant postoperative improvements were observed in ASES, VAS, and SST scores. Among the athletes included, 73% returned to sports, with the majority resuming their preinjury level of competition.

**Figure 5:**
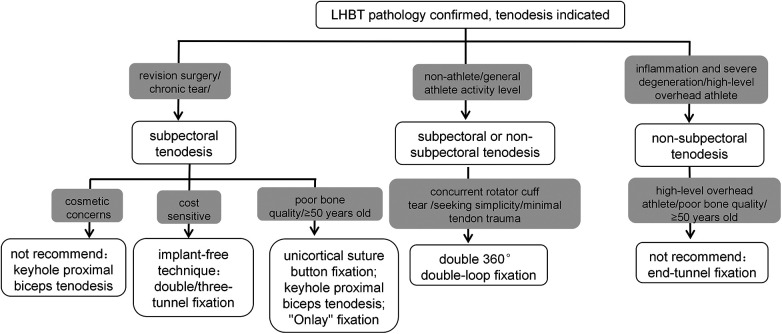
Clinical decision-making suggestions for LHBT tenodesis. Gray boxes indicate patient and clinical factors to be considered; white boxes indicate recommended techniques or techniques not recommended for use.

In summary, there are various surgical approaches for managing the LHBT, embodying the notion that “all roads lead to Rome.” In cases of primary tenodesis of the LHBT with significant inflammatory response and severe degeneration, non-subpectoral tenodesis is generally recommended. In contrast, for chronic tears or revision cases, subpectoral tenodesis is preferred. For high-level overhead athletes, non-subpectoral tenodesis (with the exception of end-tunnel fixation technique) is advised to avoid the risk of humeral fracture. Further considerations include cost sensitivity, bone quality, age, and cosmetic concerns (Figure 5). Among these techniques, the double 360° double-loop fixation stands out for its simplicity, ease of mastery, and minimal tendon trauma. It requires only a single suture anchor to achieve reliable fixation, and its suture tails can be utilized to simultaneously repair torn rotator cuffs. As such, in the authors' view, this technique holds promise as a preferred option for non-subpectoral tenodesis. Ultimately, clinicians should also consider the individual patient's specific conditions and the surgeon's experience to make personalized surgical decisions.
